# Food Waste Management Practices and Barriers to Progress in U.S. University Foodservice

**DOI:** 10.3390/ijerph19116512

**Published:** 2022-05-27

**Authors:** Aviva A. Musicus, Ghislaine C. Amsler Challamel, Robert McKenzie, Eric B. Rimm, Stacy A. Blondin

**Affiliations:** 1 Department of Social and Behavioral Sciences, Harvard T.H. Chan School of Public Health, 655 Huntington Avenue, Boston, MA 02115, USA; 2 Menus of Change University Research Collaborative, Stanford University, Stanford, CA 94305, USA; ghislaine.challamel@gmail.com; 3 Harvard College, Cambridge, MA 02138, USA; robertmckenzie@college.harvard.edu; 4 Department of Nutrition, Harvard T.H. Chan School of Public Health, 655 Huntington Avenue, Boston, MA 02115, USA; erimm@hsph.harvard.edu (E.B.R.); stacy.blondin@gmail.com (S.A.B.); 5 Department of Epidemiology, Harvard T.H. Chan School of Public Health, 677 Huntington Avenue, Boston, MA 02115, USA

**Keywords:** food waste, institutional foodservice, higher education, food insecurity, composting, food donation

## Abstract

Identifying institutional capacity to reduce and reallocate food waste is important to reduce both greenhouse gas emissions and food insecurity. The goal of this study was to examine food waste concern, reduction and repurposing strategies, and perceived barriers to these strategies among U.S. university foodservice representatives. We surveyed 57 U.S. university foodservice representatives about foodservice operations, campus food insecurity, food waste reduction and repurposing activities, and obstacles to composting and donating food waste. Data were collected September 2019–February 2020. Roughly three-quarters of respondents tracked campus food waste, reported that food waste reduction was a high/very high priority, and reported concern about campus food insecurity. The most common food-waste-reduction strategies included forecasting demand to prevent overproduction and preparing smaller batches. The most common repurposing strategies included donation and composting. Top barriers to food donation included liability concerns and lack of labor. Barriers to composting food included lack of infrastructure and knowledge/experience. Addressing perceived barriers to university foodservices’ food waste reduction and repurposing efforts could lead to reduced greenhouse gas emissions and improved food security for millions of Americans.

## 1. Introduction

Food waste is a pervasive global issue with dire implications for human and environmental health. Up to 40% of food is lost or wasted along the food supply chain [1]. From an environmental perspective, this wasted food produces up to 10% of annual global greenhouse gas emissions [1]. The United States (U.S.) is responsible for the greatest global share of greenhouse gas emissions from food waste [2]. Recapturing this edible food could not only prevent environmental harm, but provide nutrition and energy for the 38.3 million Americans living in food-insecure households [3]. Researchers have estimated that reducing and reallocating total U.S. food waste by 15% could feed ~25 million people [4] and save USD 161 billion [5].

In recognition of the severity of this issue, in 2015, the U.S. Department of Agriculture and U.S. Environmental Protection Agency announced the nation’s first-ever food-waste-reduction goal: to reduce food loss and waste by 50% by 2030 [6]. U.S. food waste occurs mainly in the later stages of the food supply chain [7], making foodservice and retail interventions crucial to achieve this goal. University campuses represent a particularly important setting to reduce food waste, as it has been estimated that, nationwide, they discard 22 million pounds of food each year [8]. This waste is driven by a variety of factors, including foodservice serving styles (e.g., buffet service) and consumer difficulties in estimating appropriate portion sizes [9,10,11]. In 2015, more than 200 campuses signed the *American Campuses Act on Climate Pledge* to “accelerate the transition to low-carbon energy while enhancing sustainable and resilient practices” [12]. Simultaneously, food-insecurity rates among U.S. college students have risen far above the national population average of 12%, with 20–50% of students reporting food insecurity [13]. Therefore, reducing and repurposing food waste on university campuses represents a promising means by which to reduce agricultural-related greenhouse gas emissions, reduce food insecurity, and save money.

Multiple studies have implemented and evaluated food-waste-reduction strategies on individual university campuses [9,14,15,16,17,18]. These studies identified several solutions that may be successful, including removing trays from the foodservice area to encourage visitors to take fewer items [15], reducing portion sizes served [9,18], and utilizing messaging campaigns [14,16]. Another previous study documented trends in food waste across institutions of higher education in 24 different countries [11]. The researchers found that the majority of surveyed universities did not have a policy on food waste, nor did they actively measure food waste [11]. Despite these studies, there is a dearth of data documenting food waste generation, awareness, and reduction initiatives at a wide range of institutions of higher education across the U.S. Such data are necessary to understand the current state of national food-waste practices and how they may need to be modified to bring the country closer to its 2030 food-waste-reduction goal. The goal of this research was to characterize and quantify food waste concern and mitigation efforts among U.S. universities, with aims to: (1) document the current landscape of food waste across U.S. institutions of higher education; (2) identify effective food-waste-reduction strategies that can be adopted by other institutions of higher education; and (3) identify common barriers universities face in mitigating food waste, and how these relate to food insecurity in their communities.

## 2. Materials and Methods

Researchers at the Harvard T.H. Chan School of Public Health developed a survey in conjunction with Harvard University Dining Services and the Harvard Food Law and Policy Clinic to profile institutions’ levels of concern about and measurement of food waste, and strategies employed/barriers experienced to reducing and repurposing food waste. The survey was administered online via Qualtrics and included questions about campus food insecurity prevalence and reduction efforts (full survey in Supplementary Material). U.S. universities that were members of university foodservice associations were recruited to take the survey (*n* = 447), including institutions in the Menus of Change University Research Collaborative (MCURC) (*n* = 51), and non-MCURC institutions that were members of the National Association of College and University Food Services (NACUFS) (*n* = 396). The MCURC is a network of colleges and universities using campus dining halls as laboratories to inform food system transformation. An invitation with a survey link was emailed to dining directors, dietitians, sustainability representatives, and professors affiliated with each institution. A reminder email was sent two weeks later. Upon providing informed consent, respondents (one per institution) were directed to the survey. The final voluntary response sample consisted of responses from 57 dining service directors/staff from 57 different universities. Data were collected September 2019–February 2020. The Harvard T.H. Chan School of Public Health Institutional Review Board approved this study. 

Descriptive statistics were calculated to summarize universities’ demographics, food service operations, food waste reduction and repurposing activities, and obstacles to composting and donating. Although the survey asked participants to quantify the average amount of total food waste at their universities, this variable was not included in the presentation of results due to limited responses and a lack of standardization in food waste measurement between universities (e.g., some estimates were reported in tons of waste, others in dollars, others in calories, etc.); meaningful descriptive statistics in this area or more complex analyses were not possible. Data were analyzed using Stata MP17 and R 3.6.3. Demographic data on U.S. geographic region, level of urbanization, undergraduate enrollment, and funding were collected from the Integrated Postsecondary Education Data System [19]. 

## 3. Results

### 3.1. Institution Characteristics

Of the responding universities (*n* = 57), 35% were from the Northeast, 30% from the West, 21% from the South, and 14% from the Midwest (Table 1). The majority were public institutions in urban areas, with institution size ranging from <5000 (21%) to >20,000 (46%). Most had self-managed dining facilities (79%). Nearly all institutions offered cook-to-serve food production, and most offered grab-n-go (81%) and all-you-care-to-eat meals (self-serve: 82%; staff-serve: 77%). 

### 3.2. Food-Waste Concern, Measurement, Reduction Efforts, and Barriers to Reduction

Seventy-two percent of respondents stated that reducing food waste was a high or very-high priority, 75% reported having made a specific goal to reduce waste, and 77% tracked waste on their campuses (Table 2). Institutions measured food waste at the pre-consumer level (47%), followed by the service level (40%), and post-consumer level (32%). One-third measured food waste at all levels (pre, post, and service), and 25% used a computer system to do so. Frequency ranged from daily, weekly, quarterly, semesterly, and annually. Thirty percent separated solid from liquid waste, and 16% separated waste into food groups. 

Institutions used many food-waste-reduction strategies (Table 2). The most common included forecasting demand to prevent overproduction (91%), preparing smaller batches (88%), using trayless dining (86%), changing menu planning (84%), and using leftovers for other dishes (81%). To repurpose food waste, 84% donated overproduced food to community partners and charitable organizations, 74% composted, 42% used food waste for industrial uses, and 12% (all rural) reused food waste as animal feed. Among schools that composted, 93% did so at the pre-consumer level and at the post-consumer level, and these institutions reported composting 72% of food waste, on average, per academic year.

The most frequent barrier to donating food was lack of labor (44%), followed by liability concerns (30%), lack of infrastructure (26%), lack of partnerships with potential recipients (21%), and state or municipal policies (19%) (Table 2). The most frequently reported barrier to composting was lack of infrastructure (58%), followed by lack of knowledge/experience with composting (44%), lack of labor (42%), and financial concerns (28%). 

### 3.3. Food Insecurity

Respondents from most institutions (74%) reported concern about campus food insecurity but only 47% reported that their institution measured it (Table 3). Resources available to help alleviate food insecurity included food banks/food pantries (60%), meal swipe donations (46%), meals offered during breaks (35%), and discounted meal plans (11%). 

## 4. Discussion

Our study surveyed foodservice directors and staff from 57 universities across the U.S. to better understand their current state of food-waste production and management, and to identify avenues for reducing and repurposing this wasted food. We found that roughly three-quarters of universities in our sample were concerned about their food-waste production, had specific food-waste-reduction goals in place, and were actively measuring their food waste. These results stand in contrast to a previous international survey of university foodservice practices, in which the majority of universities surveyed (60%) reported that they did not actively measure food waste [11]. These differences are likely due to the two studies’ drastically different samples of universities surveyed (57 universities in the U.S. vs. 52 universities in 24 countries, including the United Kingdom (*n* = 8), Malaysia (*n* = 4), and Nigeria (*n* = 4) [11]). Despite the fact that many universities in our sample reported measuring food waste, our survey revealed that food waste is not measured in a consistent way in universities across the U.S. This lack of measurement standardization presents a barrier to implementing and evaluating coordinated reduction efforts across the country and could be ameliorated through the concerted adoption of a food-waste quantification standard. One standard that could be used is the Food Loss and Waste Accounting and Reporting Standard, which was developed by a multi-stakeholder partnership, including the Food and Agriculture Organization of the United Nations and the World Resources Institute [20].

Our study also identified common strategies that U.S. universities utilize to reduce food waste, both on the production and consumer sides. On the production side, forecasting demand, preparing smaller batches of food, changing menu planning, and using leftovers for other dishes were found to be used by more than 80% of university foodservices to reduce food waste. This makes sense, given that the university dining environments included in this survey largely rotate menus every 2–5 weeks, making it more difficult for foodservice staff to inform next-day forecasting. Furthermore, the style of foodservice influences the type of waste generated, with all-you-care-to-eat environments (82% of universities surveyed) generally generating more service-level and post-consumer food waste [21]. To reduce food waste on the consumer side, we found that the majority of U.S. universities in our sample reported removing trays from the cafeteria, offering smaller portions, and using communication campaigns. Many of these solutions have been found to be effective in other university settings [9,11,14,15,16,17,18]. Studies in restaurant settings can provide insights into other ways to incentivize consumers to only take as much food as they will eat, such as instituting a pay-by-weight system and/or providing smaller containers [22].

While efforts to reduce food waste are ongoing, it is important to continue efforts to repurpose remaining wasted food. The most common repurposing method reported by universities in our sample was donation of wasted food to charitable organizations, with 84% of universities reporting utilizing this method. In contrast, only 38% of universities surveyed in an international study of food waste trends in higher education [11] reported donating food to prevent food waste [11], which highlights that institutional donations may be more difficult in countries outside of the U.S. Although donation was widespread in our sample, a main barrier was reported to be a lack of labor. Donation requires staff capacity to package and store food and manage recipient organizations. Enlisting help from student or community volunteer groups could minimize the need for extra labor. Another barrier among surveyed universities was perceived liability. Federal law provides liability protection for institutional food donations [23], so this information should be better conveyed to foodservice operators.

Increasing institutional capacity to donate wasted food that would otherwise be discarded is especially important given the high rates of food insecurity on U.S. university campuses [13]. Indeed, 74% of surveyed universities reported that food insecurity was an institutional concern. However, only 47% of universities reported measuring food insecurity rates on campus. Sixty percent of surveyed institutions reported providing food banks/pantries for food-insecure students, and nearly half offered the option for students to donate meal swipes to students in need, but providing meals during breaks and offering discounted meal plans were utilized less frequently. To directly connect students with food that would otherwise go to waste, university foodservice staff could consider using existing instructional technology to alert students when leftover food is available, as has been previously implemented and evaluated [24].

Besides food donation, the second most common repurposing method for wasted food was composting. The top barriers to composting were reported to be a lack of infrastructure, knowledge and experience, and labor. Financial concerns were the least common barrier, suggesting that upfront university investment in composting infrastructure and staff may be warranted and feasible to repurpose food waste more effectively. Leadership may also utilize existing sustainability resources [25] and turn to composting case studies from other universities [26,27] to improve their practices. They can also consult with organizations focused on food-waste reduction that provide technical assistance, such as ReFED [28] and the Harvard Food Law and Policy Clinic [29]. 

Results of this national survey can be used by foodservice operators and researchers to identify food-waste-reduction strategies and barriers. However, this study had several limitations. This sample is not representative of all universities across the country, and it lacks quantitative food-waste measurements. This prevents us from making generalizable conclusions about the association of food-waste-reduction practices and priorities with measurable outcomes. However, this study still identifies a range of strategies that U.S. universities could operationalize to reduce and repurpose food waste at their own institutions. The feasibility of different interventions will depend on each school’s circumstances. Future studies should quantify food-waste production in a standardized way across a wide range of U.S. higher education institutional foodservice operations. It will also be important to investigate COVID-19′s impact on food waste to prepare for future foodservice disruptions. 

## 5. Conclusions

Most U.S. universities surveyed in this study were concerned about their generation of food waste and were actively pursuing reduction strategies, but challenges remain to standardizing waste measurement and promoting food donation and composting. Food-waste reduction and repurposing strategies in university foodservice settings are important to reduce greenhouse gas emissions and food insecurity on campus. Addressing barriers to progress can bring the U.S. closer toward its national food-waste-reduction goal. 

## Figures and Tables

**Table 1:** Characteristics of sampled institutions (*n* = 57).

Characteristic	*n*	%
**US Region**		
Northeast	20	35%
West	17	30%
South	12	21%
Midwest	8	14%
**Level of Urbanization ^1^**		
Urban	35	61%
Rural	6	11%
Suburban	15	26%
**Funding Status**		
Public	34	60%
Private	23	40%
**Institution Size ^1^**		
0–4999	12	21%
5000–19,999	18	32%
20,000+	26	46%
**Enrollment in Undergraduate Meal Plan**		
0–4999	23	40%
5000–9999	13	23%
10,000+	8	14%
**Dining Facility Management**		
Self/Internally Operated	45	79%
Contract-Managed	10	18%
**Usage of Food Production Systems**		
Cook-to-serve	55	96%
Cook-to-order	49	86%
Assembly-serve	39	68%
Cook-to-chill	21	37%
**Types of Meals**		
All you care to eat (self-serve)	47	82%
Grab-n-go	46	81%
All you care to eat (staff-serve)	44	77%
A la carte meals	43	75%
Weighed purchase	16	28%

^1^ Institution size and Urbanization data unavailable for one institution (Institute of Advanced Study).

**Table 2:** Food-waste concern, measurement, reduction efforts, and barriers to reduction among sampled institutions (*n* = 57).

Variable	*n*	%
**Level of concern paid to addressing food waste**		
Very high	15	26%
High	26	46%
Moderate	14	25%
Low	1	2%
Not at all	0	0%
**Specific food waste goals?**		
Yes	43	75%
No	11	19%
Unsure	2	4%
**Food waste measured?**		
Yes	44	77%
No	9	16%
Unsure	1	2%
**Types of food waste measured**		
Pre-consumer	27	47%
Service	23	40%
Post-consumer	18	32%
Pre, Post, and Service combined	19	33%
Food waste measured with computer system	14	25%
**Food waste separation**		
Liquids vs. solids	17	30%
Solid food groups	9	16%
**Efforts to reduce food waste**		
Forecast demand to prevent overproduction	52	91%
Prepare smaller batches	50	88%
Trayless dining	49	86%
Change menu planning to reduce food waste	48	84%
Use leftovers for other dishes	46	81%
Offer smaller-sized plates and bowls	41	72%
Offer smaller portions	40	70%
Reduce amount of food served toward the end of the meal period	40	70%
Provide educational communications about quantity and/or impact of food waste	38	67%
Offer smaller serving utensils for self-portioned/self-served items	26	46%
Offer sample bites	17	30%
Use social norming	15	26%
**Efforts to repurpose food waste**		
Donating to charitable organizations	48	84%
Composting	42	74%
*Among composting institutions:*		
Pre-consumer level (inedible waste: plant and/or animal components that are not served/eaten)	39	93%
Post-consumer level (plate waste)	39	93%
Mean % food composted (SD)	72% (24%)	N/A
Industrial usage	24	42%
Animal feed	7	12%
**Barriers to food-waste donation**		
Labor	25	44%
Liability concerns	17	30%
Infrastructure	15	26%
Lack of recipient partnerships	12	21%
State or municipal policy	11	19%
**Barriers to Composting**		
Infrastructure	33	58%
Knowledge/experience	25	44%
Labor	24	42%
Financial concerns	16	28%

**Table 3:** Food insecurity concern and measurement among sampled institutions (*n* = 57).

Variable	*n*	%
**Is food insecurity an institutional concern?**		
Yes	42	74%
No	10	18%
**Is food insecurity measured by the institution?**		
Yes	27	47%
No	13	23%
Unsure	12	21%
**Measures to reduce food insecurity**		
Food Bank/Pantry	34	60%
Meal Swipe Donation	26	46%
Meals Available During Breaks	20	35%
Discounted Meal Plans	6	11%
Other	18	32%

## Data Availability

Data are available upon request from the authors.

## Supplementary Materials

The following supporting information can be downloaded at: https://www.mdpi.com/article/10.3390/ijerph19116512/s1. Full survey in Supplementary Materials.
