# Supplementary material for: Food Waste Management Practices and Barriers to Progress in U.S. University Foodservice
Source: Int J Environ Res Public Health. 2022 May 27;19(11):6512. doi: 10.3390/ijerph19116512 (PMC9180560; doi:10.3390/ijerph19116512)
Supplement: Supplementary file 1 [file ijerph-19-06512-s001.zip › ijerph-1700542-supplementary.pdf]

Default Question Block

Name:

First:

Last:

Preferred Email Address:

School Name (i.e. the college/university you represent):

Your Title/Position/Role:

How many undergraduates are currently enrolled at your school?

How are undergraduate dining facilities managed/operated at your school?

- Self/University-operated
- Contract-managed (e.g. Sodexo, Aramark, etc.)
-

○ [ ] Other (please describe briefly)

What type of food production system does your school use in its undergraduate dining facilities?

Please indicate the approximate percentage of meals produced by each type of service in the box to the right.

Cook-to-serve/batch cooking	
Cook-to-order	
Assembly-serve	
Cook-to-chill	
Other	

What types of meals are available in undergraduate dining facilities at your school?

Please indicate the approximate percentage each type of meal contributes to total undergraduate dining in the box to the right.

All-you-care-to-eat (staff-served/made to order)	
All-you-care-to-eat (self-served/buffet style)	
Weighed purchase (e.g. per ounce)	
A la carte meals/sides sold at a per-meal or per-item price	
Grab 'n Go (made-to-order or pre-made salads, sandwiches, etc. packaged to-go)	
Other:	
Explanation/further detail on meal type(s) selected above:	

Approximately how many undergraduates enroll in a meal plan annually (if applicable)?

Among students enrolled in a meal plan, what is the average number of meals purchased/consumed or transactions made per student each week?

How many TOTAL meals are purchased/consumed or transactions are made in your undergraduate dining facilities (including by students/staff not enrolled in a meal plan), each week?

In general, to what extent is addressing food waste on campus a concern or priority for your school/dining service provider?

	Unsure	Not at all	Somewhat	Moderately	Greatly
Extent to which food waste is a priority	○	○	○	○	○

Has your college/university made any specific goals or commitments to reducing food waste on campus?

- ○ Yes
- ○ No
- ○ Unsure

Please list any goal(s)/commitment(s) your school has made around reducing food waste (e.g. reduce total food waste by 50% by 2020, make composting available in 80% of facilities, etc.):

Please indicate how often you measure undergraduate residential dining facilities food waste at any of the following stages (e.g. 1x/semester) . Please leave blank if not measured.

Pre-consumer (food expiration/spoilage, trim waste/refuse, overproduction, overcooked, equipment failure, etc.)

Service (left on the line after meal hours)

Post-consumer (left on the tray/discarded)

Other

What is the average amount and/or value of total food waste at your college/university at each level measured?

Please provide units measured (e.g. ounces per student per day, pounds per meal, $ per week, etc.).

Please leave blank if not measured.

Pre-consumer

Service

Post-consumer

Other

Please indicate at which meal period(s) food waste is measured:

Breakfast

Lunch

Dinner

Other

When measuring food waste, are solid foods and beverages (e.g. milk, soda, water) measured separately?

- ○ Yes
- ○ No
- ○ ______ Other

What is the average amount of liquid (beverage) waste at your college/university at each level measured? Please provide units measured (e.g. ounces per student per day).

Production	
Service	
Consumer	
Other	

When measuring solid food waste, do you separate waste by food group (e.g. meat, dairy, fruits, vegetables, grains)?

- ○ Yes
- ○ No
- ○ ______ Other

Please briefly describe the methods you use to measure food waste by food group:

______

Does your school engage in any of the following production- or consumer-level food waste reduction efforts in its undergraduate facilities? Please select all that apply.

- ☐ Forecast demand to prevent overproduction
- ☐

Prepare smaller batches

- ☐ Offer sample bites
- ☐ Offer smaller portions
- ☐ Offer smaller-sized plates and bowls (either in addition to or instead of larger options)
- ☐ Offer smaller serving utensils for self-portioned/self-served items (e.g. smaller scoops, ladles)
- ☐ Use social norming (e.g. clean plate club)
- ☐ Provide educational communications about quantity and/or impact of food waste
- ☐ Trayless dining
- ☐ Change menu planning to reduce food waste (e.g. remove unpopular items, reduce variety, etc.)
- ☐ Reduce amount of food served toward the end of the meal period
- ☐ Use leftovers for other dishes
- ☐ ________ Other:
- ☐ ________ Other:

Does your school engage in any of the following to repurpose food waste? Please select all that apply.

- ☐ Donate to charitable organizations (e.g. food banks, soup kitchens, shelters, etc.)
- ☐ Animal feed
- ☐ Composting
- ☐ Industrial usage (e.g. fuel conversion)
- ☐ ________ Other (please describe briefly):
- ☐ Not currently.

Please provide a brief explanation of how your campus repurposes food waste as animal feed:

____

Please provide a brief explanation of how your campus repurposes food waste for industrial usage:

At what level(s) do you compost food waste?

- ○ Pre-consumer (inedible waste: plant and/or animal components that are not served/eaten)
- ○ Post-consumer (plate waste: leftover food, fruit peels, etc.)
- ○ Both (pre- and post-consumer)
- ○ Other:

If known, please approximate the percentage of food waste is composted on average per academic year:

	0	10	20	30	40	50	60	70	80	90	100
Percentage of food waste composted											

Are compost bins offered in locations on campus other than undergraduate dining facilities?

- ☐ Dining facilities only
- ☐ Other locations on campus:
- ☐ Other or further explanation:

How is consumer compost (i.e. plate waste) sorted from non-compostable items? Please select all that apply.

- ☐ Students sort their own plate waste into trash/compost receptacles
- ☐ Food service staff sort students' plate waste into trash/compost receptacles
- ☐ ____ Other

Would you like to compost food waste at your college/university?

- ○ Yes
- ○ No
- ○ Maybe

Please list any challenges or barriers to composting food waste at your college/university (e.g. financial, space, expertise, etc.):

- ○ Financial
- ○ Space
- ○ Labor constraints
- ○ Knowledge/training/expertise
- ○ ____ Other
- ○ ____ Other
- ○ Explanation/further detail on challenges/barriers selected ____
- ○ None of the above

To your knowledge, does your city or state have any policies or incentives in place to mandate or encourage composting?

- ○ Yes
- ○ No

- Don't know or not sure
- [ ] Comments

What type(s) of plate ware/utensils are available in your dining facilit(ies)?

Please approximate the percentage each type constitutes of the total utensils used.

Reusable (e.g. china, plastic, or synthetic)

Compostable

Recyclable

Disposable

Other:

Comments/further detail:

Please provide additional details on your food donation efforts (e.g. who receives the food, what kind of partnership do you have with recipients, what and how much do you donate, who manages the process, etc.):

Who receives the food you donate?

Is the food distributed directly or by a third party?

How much food do you donate annually?

Who manages the food donation process?

Is your college/university interested in donating leftover, excess, or food that is otherwise not consumed?

- Yes
- No
- Maybe
- Don't know/Unsure

Please list any challenges or barriers to donating food at your college/university (e.g. staffing, recipient partnerships, laws/policies, liability, etc.):

- Labor/staff constraints
- Recipient partnership(s)
- State or municipal laws/policies
- Liability concerns
- ______ Other
- ______ Other
- Explanation/further detail on any challenges/barriers selected ______
- None of the above

Is food insecurity a concern among students at your college/university?

Note: Food insecurity is defined as the lack of consistent access to enough food for an active, healthy life.

- Yes
- No
- Don't Know/Unsure

Does your college/university measure food insecurity among enrolled students?

- Yes
- No
- Don't Know/Unsure

Which of the following, if any, are available at your college/university to reduce student food insecurity? Please select all that apply.

Food bank/pantry

- Donation of meal swipes
- Discounted meal plans for low-income students
- Other
- Other

Please briefly describe the methodology you used to measure food insecurity and any results you are able/willing to share.

Please share any additional comments on food waste, including mitigation strategies, goals/plans, and/or ideas, at your college/university:

If you would like to upload any relevant files or images, please do so here:

Powered by Qualtrics
